# Identification of biological pathways and processes regulated by NEK5 in breast epithelial cells via an integrated proteomic approach

**DOI:** 10.1186/s12964-022-01006-y

**Published:** 2022-12-22

**Authors:** Camila de Castro Ferezin, Terry C. C. Lim Kam Sian, Yunjian Wu, Xiuquan Ma, Anderly C. Chüeh, Cheng Huang, Ralf B. Schittenhelm, Jörg Kobarg, Roger J. Daly

**Affiliations:** 1 Cancer Program, Biomedicine Discovery Institute, Monash University, Melbourne, VIC 3800 Australia; 2 Department of Biochemistry and Molecular Biology, Monash University, Melbourne, VIC 3800 Australia; 3 Faculty of Pharmaceutical Sciences, State University of Campinas, São Paulo, Brazil; 4 Monash Proteomics and Metabolomics Facility, Monash University, Melbourne, VIC 3800 Australia

**Keywords:** NEKs, NEK5, Kinase, Breast cancer, Proteomics, BioiD

## Abstract

Specific members of the Nima-Related Kinase (NEK) family have been linked to cancer development and progression, and a role for NEK5, one of the least studied members, in breast cancer has recently been proposed. However, while NEK5 is known to regulate centrosome separation and mitotic spindle assembly, NEK5 signalling mechanisms and function in this malignancy require further characterization. To this end, we established a model system featuring overexpression of NEK5 in the immortalized breast epithelial cell line MCF-10A. MCF-10A cells overexpressing NEK5 exhibited an increase in clonogenicity under monolayer conditions and enhanced acinar size and abnormal morphology in 3D Matrigel culture. Interestingly, they also exhibited a marked reduction in Src activation and downstream signalling. To interrogate NEK5 signalling and function in an unbiased manner, we applied a variety of MS-based proteomic approaches. Determination of the NEK5 interactome by Bio-ID identified a variety of protein classes including the kinesins KIF2C and KIF22, the mitochondrial proteins TFAM, TFB2M and MFN2, RhoH effectors and the negative regulator of Src, CSK. Characterization of proteins and phosphosites modulated upon NEK5 overexpression by global MS-based (phospho)proteomic profiling revealed impact on the cell cycle, DNA synthesis and repair, Rho GTPase signalling, the microtubule cytoskeleton and hemidesmosome assembly. Overall, the study indicates that NEK5 impacts diverse pathways and processes in breast epithelial cells, and likely plays a multifaceted role in breast cancer development and progression.

Video Abstract

**Supplementary Information:**

The online version contains supplementary material available at 10.1186/s12964-022-01006-y.

## Background

Breast cancer is one of the leading causes of death worldwide and is therefore a major public health concern [1]. It is a highly heterogeneous disease composed of various subtypes that vary according to molecular and histopathological features and clinical behaviour and can be classified based on gene expression patterns into luminal A and B, HER2 and triple negative subgroups [2]. Eventual metastatic spread to distant organs—predominantly bones, lungs, and brain—represents a significant clinical problem and represents the primary cause of death for the majority of patients [3]. Breast cancer emerges as a consequence of subgroup-selective dysregulation of particular signalling pathways in mammary epithelial cells, leading to particular interest in identification of pathways susceptible to targeted therapeutic intervention. Since protein kinases are fundamental players in the molecular mechanisms underlying cell signalling and their deregulation leads to development and progression of various types of cancer [4], they have been pursued as key therapeutic targets. For example, the receptor tyrosine kinase HER2 is targeted by the monoclonal antibody therapy trastuzumab in HER2 breast cancers [5], CDK4/6 by palbociclib in endocrine therapy-resistant luminal breast cancers [6] and particular FGFR family members represent potential therapeutic targets in subsets of luminal and triple negative breast cancer patients [7].

Despite the fact that specific protein kinases represent some of the most well-known targets for cancer treatment, a considerable proportion of the protein kinase family, often referred to as the ‘dark kinome’, remains poorly characterized and warrants further investigation [8, 9]. Nima Related Kinases (NEKs) are a family of Serine/Threonine kinases that are in general understudied and have been subjected to little targeted medicinal chemistry effort, despite several NEKs being linked to tumorigenesis or cancer progression [10–17]. Out of the 11 members of the NEK family, NEK5 is one of the least characterized, both structurally and functionally. Recent publications have demonstrated the association of NEK5 with poor prognosis and tumour progression in breast cancer [18, 19]. However, the different functions of NEK5 in both normal and cancer cells remain poorly understood.

Here, we established a new model system for NEK5 in breast cancer, demonstrating that NEK5 overexpression in MCF-10A immortalized human mammary epithelial cells enhanced clonogenicity and led to aberrant growth in 3D culture. Moreover, interrogation of this model via an integrated proteomic approach involving definition of the NEK5 interactome by BioID and global (phospho)proteomics revealed novel functions for this kinase and unsuspected impact on known oncogenic signalling pathways.

## Methods

### DNA vectors

For BioID, the mycBioID2-pBABE-puro vector was obtained from Addgene (Plasmid #80900) and NEK5 (wild type) was amplified from pcDNA5.1-NEK5^WT^ [20] and subcloned into mycBioID2-pBABE-puro.

### Cell culture

MCF-10A cells stably expressing the murine ecotropic receptor (MCF-10A EcoR) were used to generate stable pools of MCF-10A cells expressing NEK5-BioID (referred as to MCF-10A NEK5) or control vector (referred as MCF-10A EV) [21]. To produce stable cell lines, PlatE cells were transfected with DNA vectors using Lipofectamine 3000 (Invitrogen) according to the manufacturer’s instructions. Viral supernatants were collected at 48 h and 72 h after transfection and filtered through 0.45 μm pore-size filter caps (Millipore). MCF-10A EcoR cells were infected with viral supernatants for 24 h in the presence of 8 μg/ml polybrene (Millipore). Successfully transduced cells were selected with puromycin (2 μg/mL) for pBABE-BioID constructs. MCF-10A cells and their derivatives were maintained in Dulbecco’s modified Eagle’s medium/nutrient mixture F-12 (Invitrogen) supplemented with 5% (v/v) horse serum (Invitrogen), 20 ng/ml human recombinant EGF (R&D Systems), 0.5 μg/ml hydrocortisone (Sigma), 100 ng/ml cholera toxin (Sigma), and 10 μg/ml bovine insulin (Sigma).

### Immunoblot

For the extraction of total cellular protein, cells were washed with ice-cold PBS and lysed with lysis buffer (50 mM Tris–HCl [pH 7.4], 150 mM NaCl, 1 mM EDTA [pH 7.4], 1% NP-40 with protease and phosphatase inhibitors cocktail) treated for 5 min at 96 °C and centrifuged at 20,000 × g for 15 min. Protein concentrations were estimated by Pierce BCA protein assay (Thermo Fisher Scientific). The whole-cell lysate was mixed with a 1/5 volume of 5 × SDS sample buffer and boiled. Total cell proteins were resolved by SDS-PAGE followed by electro-transfer of proteins onto a PVDF membrane. The membrane was blocked for 1 h at room temperature (RT) using BSA/Tris buffered saline with 0.05% Tween 20 (TBST). The membrane was then incubated with primary antibodies overnight at 4 °C. After washing with TBST 3 times for 5 min, the membrane was incubated with horseradish peroxidase-conjugated anti-rabbit IgG or anti-mouse IgG at 1:5000 (Santa Cruz Biotechnology, Santa Cruz, CA, USA) for 1 h at RT. After washing with TBST 3 times for 5 min, blots were developed using the enhanced chemiluminescence ECL Western Blotting System (Amersham).

### Antibodies

NEK5 (HPA041399) was purchased from Sigma. The following antibodies were purchased from CST: Src (2123), pSrc (6943), FAK (3285), pFAK (3281), Paxillin (2542), pPaxillin (69,363), Akt (4685), pAkt (4058), 14-3-3 (8312), pErk1/2 (4370), Erk1/2 (4695), MCAM (81,701) and NCAPD3 (13,473). The β-actin antibody was from MP Biomedicals (691,001).

### BioID

MCF-10A cells expressing NEK5-BioID or BioID Empty Vector (EV) were cultivated in 15 cm dishes until reaching 70% confluency and treated with a final concentration of 50 μM of Biotin for 18 h. Cells were washed twice with ice-cold PBS and harvested in 800 μL of modified RIPA-Buffer (50 mM Tris–HCl pH 7.5, 150 mM NaCl, 1 mM EDTA, 1 mM EGTA, 1% Triton X-100, 0.1% SDS supplemented with protease inhibitor cocktail and 250U Turbonuclease). Cell lysates were then transferred to a 15 mL conical tube and sonicated for two sessions with 30 pulses using a Branson Sonifier at 30% duty cycle and an output level of 3. Tubes were left on ice for 1 min between each session to prevent overheating and spun down 30 min at 16,500 × g at 4 °C. Cleared supernatants were transferred to a new 15-ml conical tubes and BCA was performed to measure protein concentration. Equivalent amounts of protein lysates were transferred to new 15-ml conical tubes and the volume adjusted with lysis buffer. Streptavidin agarose beads were washed 3 times with 1 ml modified-RIPA buffer (minus protease inhibitor cocktail and Turbonuclease), then 50 μl bead volume of pre-washed Streptavidin agarose beads were transferred to each sample and incubated on a rotator at 4 °C for 3 h. The beads were washed with modified RIPA buffer then 50 mM ammonium bicarbonate (NH4HCO3) pH 8. Trypsin digestion was then performed at 37 °C overnight with agitation. The supernatants were then transferred to fresh 1.5 ml tubes, the remaining beads were rinsed 2 times with mass spec-grad H_2_O and these rinses were combined with the original supernatant. Following lyophilization in a speed-vac, the samples were resuspended in 0.1% formic acid and the peptide concentration measured using a Nanodrop. Finally, the concentrations of all samples were normalized and analysed by Mass Spectrometry (MS; Q-Exactive Plus Hybrid Quadrupole-Orbitrap from Thermo Scientific) in the Monash Proteomics & Metabolomics Facility (MPMF). Data were analysed using MaxQuant to obtain protein identifications and their respective label-free quantification (LFQ) values using in-house standard parameters.

### Colony formation assay

Cells were plated in 12-well plates at 200 cells/well. Media was replaced every 3 days until colonies were evident, after 7–8 days. Cells were then fixed with methanol and colonies stained with 0.4% crystal violet. Colony number and colony size were quantified with ImageJ.

### Three-dimensional culture of MCF-10A cells on Matrigel™

Cells were trypsinized and resuspended in 3D no-EGF medium (Dulbecco’s modified Eagle’s medium/nutrient mixture F-12 supplemented with 2% (v/v) horse serum, 100 ng/ml cholera toxin, 0.5 μg/ml hydrocortisone, 10 μg/ml bovine insulin) or 3D + EGF medium (Dulbecco’s modified Eagle’s medium/nutrient mixture F-12 supplemented with 2% (v/v) horse serum, 100 ng/ml cholera toxin, 0.5 μg/ml hydrocortisone, 10 μg/ml bovine insulin, 5 ng/ml EGF), and plated into a 96-well plate pre-coated with 40 μl of Matrigel at a density of ~ 800 cells/well in medium containing 2% Matrigel. Cells were allowed to form acini for up to 12 days and fresh medium was replaced every 3 to 4 days. The plate was evaluated by overlay confocal imaging and analysed using LAS-F (Leica Microsystems^©^) and Fiji ImageJ.

For roughness evaluation we used Fiji ImageJ Software. The circular perimeter (Pconvex) of each spheroid was measured using the circular tool, followed by the measurement of the roughness perimeter (real area). For each of the biological replicates, two wells were evaluated and at least 15 random acini were analysed.

### Mass spectrometry-based proteomics

MCF-10A -NEK5 and control cells were cultivated in 15 cm dishes until they reached 80% confluency. Cells were washed with ice-cold TBS twice and harvested with SDC lysis buffer (4% sodium deoxycholate, 100 mM Tris–HCL pH 8.5) and immediately heat-treated at 95 °C for 5 min. Lysates were homogenised by sonication, and an aliquot was taken for BCA assay. Disulphide bonds and carbamidomethylate cysteine residues were reduced using Reduction/Alkylation Buffer (100 mM Tris(2-carboxyethyl)phosphine (TCEP) and 400 mM 2-chloroacetamide pH 7) and heated at 95 °C for 10 min. Samples were allowed to cool and then subjected to Trypsin and LysC digestion (2 µg of each enzyme per 200 µg of protein) overnight at 37 °C with shaking. The SDC was precipitated out of solution with 400 µL of isopropanol (ISO) and 100 µL EP enrichment buffer (48% trifluoroacetic acid (TFA) and 8 nM KH_2_PO_4_) was added to each sample which were then centrifuged to clear the supernatants (2000 × g, 15 min). From this step, we separated the samples for Whole Proteome and Phosphoproteome analysis.

### Sample preparation for whole proteome

Following centrifugation, supernatants were collected, and a desalting step was performed using C18 spin columns as previously described in [22]. After elution, samples were dried using a SpeedVac and then resuspended in 2% (v/v) acetonitrile (ACN)/0.1% (v/v) formic acid (FA) to a peptide concentration of 0.1 µg/µL.

### Sample preparation for phosphoproteome

Following centrifugation, supernatants were transferred to a clean 96-well plate. Phosphopeptides were enriched as previously described [23]. Briefly, peptides were enriched with a 12:1 TiO_2_ bead (5010-21315, GL Sciences, Tokyo, Japan) to protein ratio for 5 min at 40 °C with shaking (2000 rpm). Phosphopeptides were eluted with EP elution buffer (5% (v/v) NH_4_OH in 32% (v/v) ACN) prior to desalting with in-house prepared SDB-RPS (Empore™, CDS Analyticl, Oxford, PA, USA) stage tips and eluted with 20 μl of 25% (v/v) NH_4_OH in 60% (v/v) ACN and evaporated to dryness in a SpeedVac. The dried peptides were reconstituted in 2% (v/v) ACN /0.3% (v/v) TFA.

### Mass spectrometry analysis

Samples were analyzed on a UltiMate 3000 RSLC nano LC system (Thermo Fisher Scientific) coupled to a Q Exactive HF mass spectrometer (Thermo Fisher Scientific). Peptides were loaded via an Acclaim PepMap 100 trap column (100 μm × 2 cm, nanoViper, C18, 5 μm, 100 Å, Thermo Fisher Scientific) and subsequent peptide separation was on an Acclaim PepMap RSLC analytical column (75 μm × 50 cm, nanoViper, C18, 2 μm, 100 Å, Thermo Fisher Scientific). For each liquid chromatography-tandem mass spectrometry (LC–MS/MS) analysis, 1 μg of peptides as measured by a nanodrop 1000 spectrophotometer (Thermo Fisher Scientific) was loaded on the pre-column with microliter pickup. Peptides were eluted using a 2 h linear gradient of 80% (v/v) ACN/0.1% FA at a flow rate of 250 nL/min using a mobile phase gradient of 2.5–42.5% (v/v) ACN. The eluting peptides were interrogated with an Orbitrap mass spectrometer. The HRM DIA method consisted of a survey scan (MS1) at 35,000 resolution (automatic gain control target 5e6 and maximum injection time of 120 ms) from 400 to 1220 m/z followed by tandem MS/MS scans (MS2) through 19 overlapping DIA windows increasing from 30 to 222 Da. MS/MS scans were acquired at 35,000 resolution (automatic gain control target 3e6 and auto for injection time). Stepped collision energy was 22.5, 25, 27.5% and a 30 m/z isolation window. The spectra were recorded in profile type. For peptide identification, the false discovery rate (FDR) was set to 1% at peptide level. The raw data files were analysed with the MaxQuant Version 1.6.0.16 analysis software using default settings.

### Mass spectrometry statistical analysis

Peptide intensities were Log_2_ transformed, imputation was via normal distribution with Perseus software before quantile normalization. The comparison between NEK5 overexpressing cells and control cells was assessed by corresponding fold change (FC) and a two-tailed *t*-test with a *p* < 0.05.

### Bioinformatics analysis and data mining

Functional annotation and pathway analysis of the interactome and (phospho)proteome were conducted using Metascape [24]. Overrepresented functional categories among proteins enriched (FC > 2 for NEK5 interactome with adjusted *p* < 0.05; FC > 1.5 for proteins/phosphosites in NEK5 overexpressing cells with *p* < 0.05) were evaluated using hypergeometric distribution with Benjamini Hochberg corrected p value (*p* < 0.01). Criteria for reported functional enrichment required a p value < 0.01, FDR < 0.05 and > 3 proteins mapping to a functional pathway. Experimentally verified and published protein–protein interactions from STRING [25] and Cytoscape [26] were assessed. RNA-seq data deposited in the Cancer Cell Line Encyclopedia (CCLE) and Cancer Dependency Map (DepMap) databases were used to determine the mRNA expression of NEK5 and its interacting proteins in human breast cancer cell lines and the immortalized breast epithelial cell line, HMEL.

### Statistical analysis

Student’s t-test followed by Bonferroni post-hoc test was used for statistical analyses. The data in this study are presented as the mean and error bars represent the standard deviation. The data were acquired from at least three independent experiments. *p < 0.05 was considered significant differences among the experimental groups. The software used was GraphPad Prism version 8 (GraphPad Software, La Jolla California USA, www.graphpad.com).

## Results

### Establishment of a model system for interrogation of NEK5 function in breast cancer

**Fig. 1:**
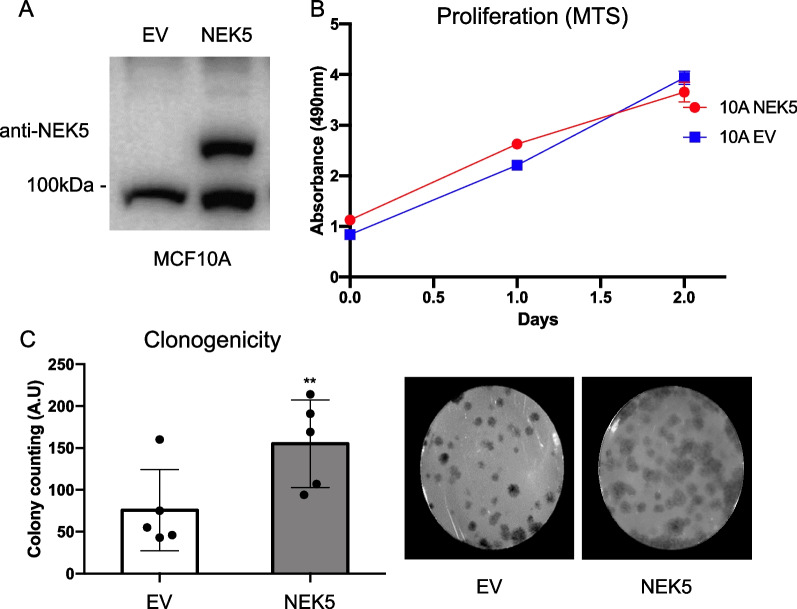
Effect of NEK5 overexpression in MCF-10A cells. **A** Expression of NEK5-BioID-MYC vector in MCF-10A cells. Cell lysates were Western blotted with an anti-NEK5 antibody. EV- empty vector. **B** Effect of NEK5 overexpression on monolayer proliferation. Cells were subjected to a MTS assay. Data represent the mean and standard deviation (SD) of 5 biological replicates. **C** Effect of NEK5 overexpression on clonogenicity. MCF-10A NEK5 and control cells were plated at low-density and incubated for 8 d prior to colony quantification. The data represent the mean and SD of 5 replicates. *Indicates *p* < 0.05

Emerging evidence supports a role for NEK5 in breast cancer development and progression [18, 19]. In order to interrogate NEK5 function in breast cancer and the NEK5 signalling mechanism in breast epithelial cells via Bio-ID proteomics (Additional file 1: Fig. S1A), we established MCF-10A immortalized mammary epithelial cells expressing a NEK5-BirA fusion protein (Fig. 1A). We selected this model because three-dimensional culture of MCF-10A mammary epithelial cells on a reconstituted basement membrane results in formation of polarized, growth-arrested acini-like spheroids that recapitulate several aspects of glandular architecture in vivo. Oncogenes introduced into MCF-10A cells disrupt this morphogenetic process, and elicit distinct phenotypes [27]. Western blotting detected a protein of the expected size for the NEK5-BirA fusion expressed at similar levels to endogenous NEK5, indicating that NEK5 was overexpressed approximately twofold in these cells (Fig. 1A).

**Fig. 2:**
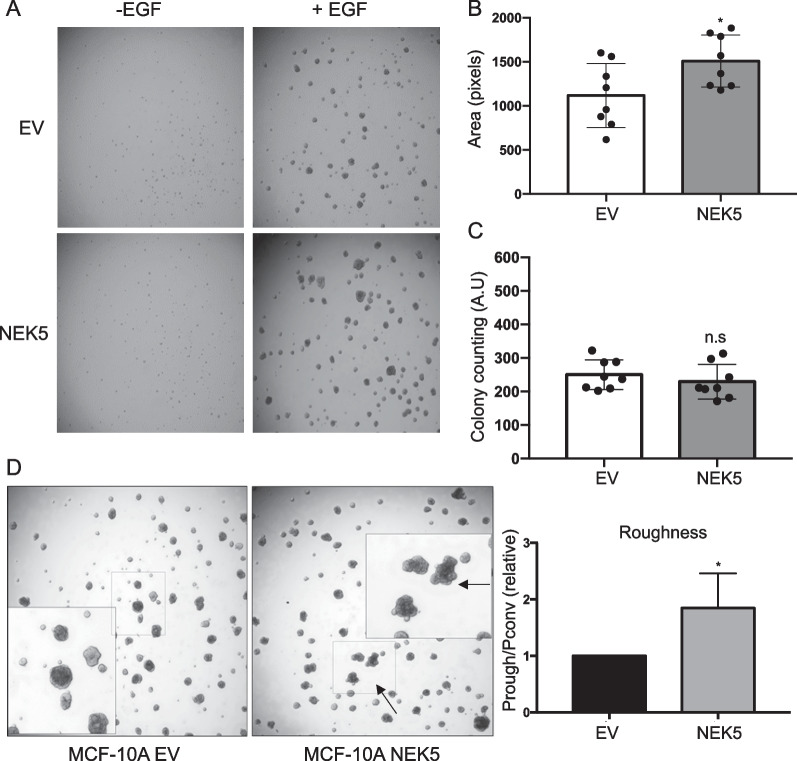
Effect of NEK5 on MCF-10A acinar growth and morphology. **A** Effect on acinar growth. Images are of acini grown in the presence or absence of EGF and acinar size (**B**) and number (**C**) quantified by Image J software. **D** Effect on acinar morphology. ‘Rough’ acini with protrusions were quantified. The data represent the mean and SD of 8 replicates. *Indicates *p* value < 0.05, ns indicates not significant

First, we indirectly evaluated anchorage-dependent proliferation rates of MCF-10A cells overexpressing NEK5 by MTS assay, as well as clonogenicity ie the ability of cells plated at low density to form colonies. While cell proliferation in monolayer was not affected by NEK5 overexpression (Fig. 1B), a significant increase in colony formation was observed (Fig. 1C). Next, we determined the impact of NEK5 overexpression on acinar growth in 3D culture in either the absence or presence of exogenous EGF. While neither the control or NEK5-overexpressing cells could form acini in the absence of EGF, NEK5 overexpression led to a significant increase in acinar size but not acinar number (Fig. 2A–C). We also observed that MCF-10A acini overexpressing NEK5 exhibited acinar protrusions on the cell membrane (Fig. 2D), therefore, we evaluated the roughness of the acini by measuring their average circular perimeter (Pconvex) and the rough perimeter (Prough). This quantitative analysis confirmed the impact of NEK5 on acinar morphology (Fig. 2D).

**Fig. 3:**
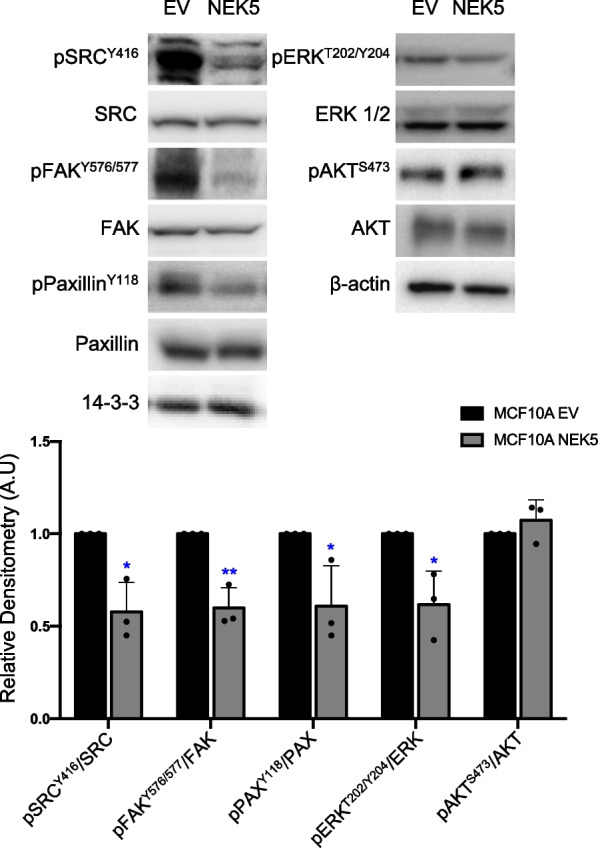
Effect of NEK5 on cell signaling pathways in MCF-10A cells. Cell lysates were Western blotted as indicated. The histograms indicate the phosphosignal normalized for total levels of the corresponding protein. The data represent the mean and SD of 3 independent experiments. *p < 0.05; **p < 0.01

Next, we determined the effect of NEK5 on signalling pathways associated with enhanced proliferation and altered morphology of MCF-10A cells in 3D culture, specifically MEK/Erk, PI3-kinase/Akt, and Src [21, 28, 29]. Western blot analysis revealed that NEK5 overexpression did not affect activation of Akt and was associated with significantly decreased activation of Erk (Fig. 3). In addition, activation of Src, and tyrosine phosphorylation of its downstream targets FAK and paxillin, were also significantly decreased.

Overall, our data demonstrated that NEK5 overexpression in MCF-10A cells leads to changes in clonogenicity in 2D and both acinar growth and morphology in 3D, establishing these cells as an interesting and powerful model to characterize NEK5 mechanism and function. However, the counterintuitive effect of NEK5 on canonical signalling pathways in these cells emphasized the need for an unbiased approach to characterize NEK5 signalling, involving determination of the NEK5 interactome and the impact of NEK5 on cell signalling networks.

### Determination of the NEK5 interactome in MCF-10A cells using BioID

**Fig. 4:**
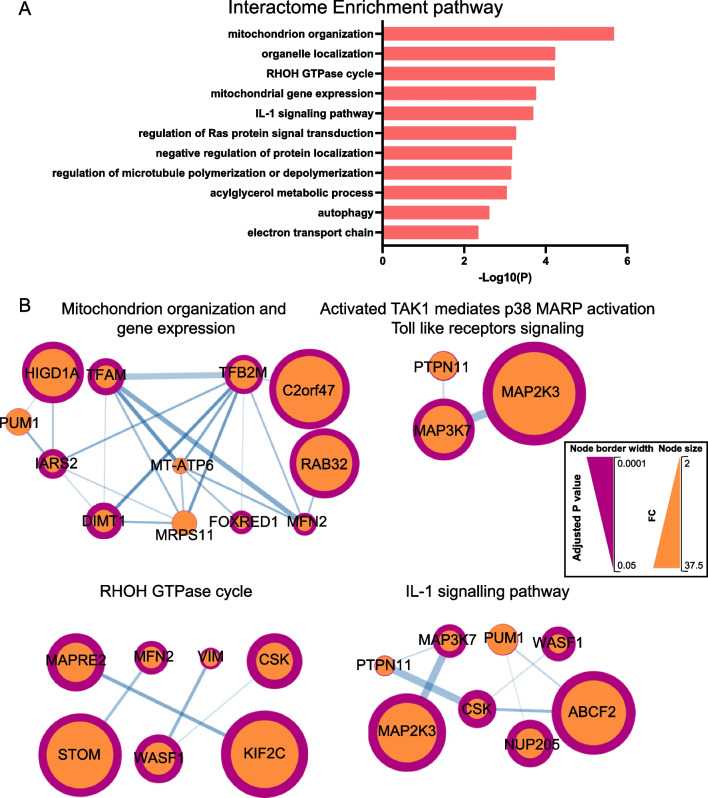
Functional analysis of NEK5 interactors identified by BioID. **A** Cell signaling pathway enrichment of NEK5 biotinylated proteins. Data were analysed using Metascape [24]. **B** Major protein interaction networks in the NEK5 interactome. Analysis was undertaken using STRING/Cytoscape. The node size indicates the fold change (FC) for biotinylation in NEK5-BioID-MYC-expressing cells versus control and the node border width indicates the *p* value. Proteins with higher FC and lower p value are represented by larger nodes with wider border

NEK5 is one of the least understudied members of the NEK family. It has been linked mostly to centrosome disjunction and cell-cycle regulation [30], but it also has emerging roles in apoptosis, muscle differentiation [31], mitochondrial metabolism and mtDNA maintenance [20, 32]. However, an integrated proteomic analysis, with the power to identify interactors and downstream pathways, has yet to be undertaken for NEK5. In the BioID approach, the protein of interest is fused to a promiscuous biotin ligase (BirA), expressed in living cells, and allowed to biotinylate proximal proteins during a defined labelling period by biotin supplementation [33] (Additional file 1: Fig. S1A). By expressing a NEK5-BirA fusion in MCF-10A cells (Fig. 1A) and applying the BioID workflow (Additional file 1: Fig. S1A) we identified biotinylated proteins significantly enriched in cells expressing the NEK5-BirA fusion (Additional file 1: Fig. S1B, Additional file 2: Table S1). Bioinformatic analysis of the interactors in terms of cellular functions and pathways, and interactions, reinforced the role of NEK5 in mitochondria [20, 32], with ‘Mitochondrion organization’, ‘Mitochondrial gene expression’ and ‘electron transport chain’ included in the significantly enriched functional categories (Fig. 4A). In this context, TFAM and TFB2M (both components of the mitochondrial transcription initiation complex) and CLPX, a component of a mitochondrial protease that regulates TFAM, were detected as NEK5 interactors, implicating NEK5 in mitochondrial DNA maintenance, transcription and repair (Fig. 4A, B, Additional file 1: Fig. S1B, Additional file 2: Table S1) [34–38]. In addition, the enrichment for ‘regulation of microtubule polymerization or depolymerization’, reflecting the presence of KIF2C and KIF22, kinesin-like proteins that function in mitotic chromosome segregation, as well as MAPRE2, a microtubule-associated protein that regulates mitotic spindle formation [39–41], was consistent with known mitotic roles of NEK5, including regulation of centrosome function and chromosomal segregation [30] (Fig. 4A). However, an interesting finding was the enrichment for the RhoH GTPase cycle, which appears to reflect the presence of cytoskeletal regulators rather than RhoH itself, which is specific to the hematopoietic system [42].

The BioID approach also identified a variety of signalling proteins as NEK5 interactors. These included: MAP2K3 and MAP3K7, two serine/threonine kinases that participate in signalling cascades initiated by cytokine and stress stimuli [43, 44]; the non-receptor tyrosine kinase CSK, a negative regulator of Src family kinases [45]; the non-receptor protein tyrosine phosphatase PTPN11 [46]; and the scaffold protein SHC1, which orchestrates cellular responses to growth factor signals [47]. Reflecting the presence of these proteins, pathways and interaction networks associated with inflammatory signalling and regulation of Ras were enriched in the NEK5 interactome (Additional file 1: Fig. S1B, Fig. 4A, B).

We also determined to what extent the identified NEK5 interactors are co-expressed with NEK5 in breast cancer. Interrogation of publically-available gene expression data revealed that while the mean expression of NEK5 across breast cancer cell lines is not significantly higher than in the human immortalized breast epithelial cell line HMEL, a subset of the breast cancer cell lines exhibit markedly higher expression than HMELs (Additional file 1: Fig. S2A). In addition, expression of NEK5 is significantly higher in ER-positive cell lines than ER-negative ones (Additional file 1: Fig. S2A). Therefore, we focused our co-expression analysis to the ER-positive lines. This identified that while mean NEK5 expression is relatively low compared to its interactors, all of the interactors are co-expressed with NEK5 in ER-positive breast cancer cell lines, and at varying levels (Additional file 1: Fig. S2B). This supports the relevance of the identified NEK5 interactome to breast cancer development and progression.

### Phosphoproteomic analyses and data integration reveal new biological processes regulated by NEK5 kinase

**Fig. 5:**
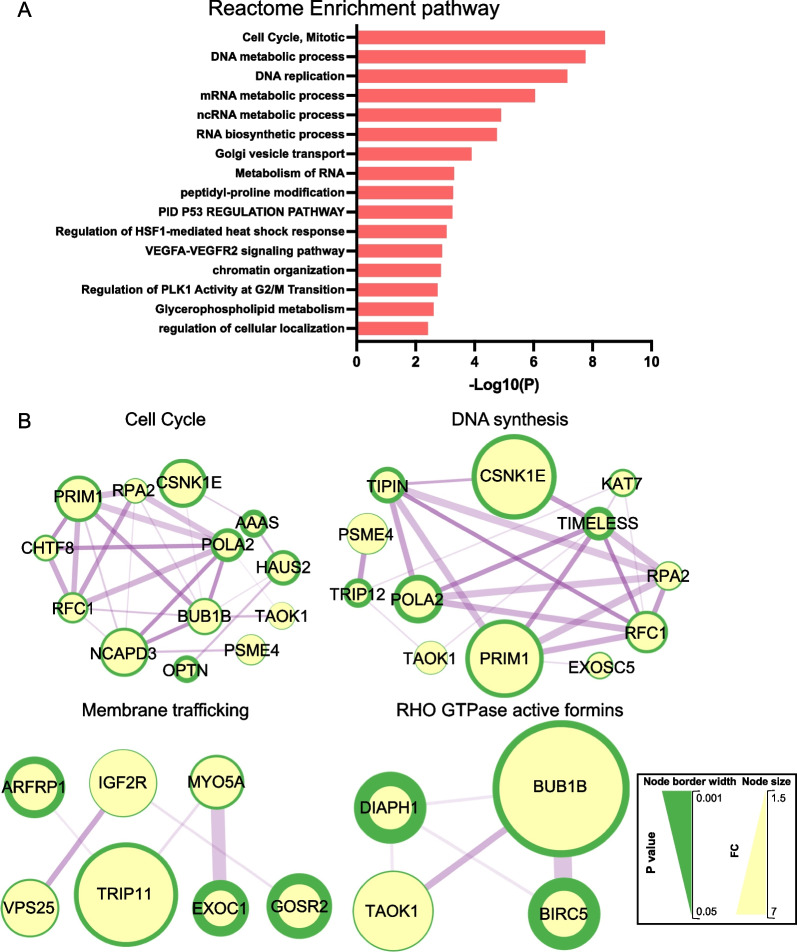
Characterization of proteomic changes in NEK5-overexpressing cells. **A** Functional categories associated with proteins with increased abundance in NEK5-overexpressing cells. Data were analysed using Metascape [24]. **B** Major protein interaction networks formed by proteins with increased abundance in NEK5-overexpressing cells. Analysis was undertaken using STRING/Cytoscape. Explanation of scales is provided in Fig. 4 legend

To gain further insights into NEK5 signalling, we first characterized the impact of NEK5 on the total proteome and phosphoproteome (Additional file 2: Tables S2, S3). Key proteins exhibiting significantly enhanced or decreased protein expression are summarized in the volcano plot in Additional file 1: Fig. S3. A striking finding was enrichment for proteins involved in cell cycle regulation and DNA metabolism/synthesis amongst those increased upon NEK5 overexpression (Fig. 5A, B). Regarding cell cycle control, key proteins identified were BUB1B, a spindle checkpoint kinase, and NCAPD3, a protein that regulates mitotic chromosome assembly and segregation (Fig. 5B), while for DNA synthesis, important proteins included subunits of DNA polymerase alpha (POLA2), DNA primase (PRIM1) and replication factor C (RFC1) (Fig. 5B). In addition, an enrichment for vesicle transport and membrane trafficking, as well as Rho GTPase signalling, was also observed (Fig. 5A, B), the latter of interest given the presence of a RhoH-associated network in the NEK5 interactome (Fig. 4B). Western blot analysis confirmed the increased expression of NCAPD3, and decreased expression of MCAM, in the NEK5-overexpressing cells, providing validation of the MS data (Additional file 1: Fig. S4).

**Fig. 6:**
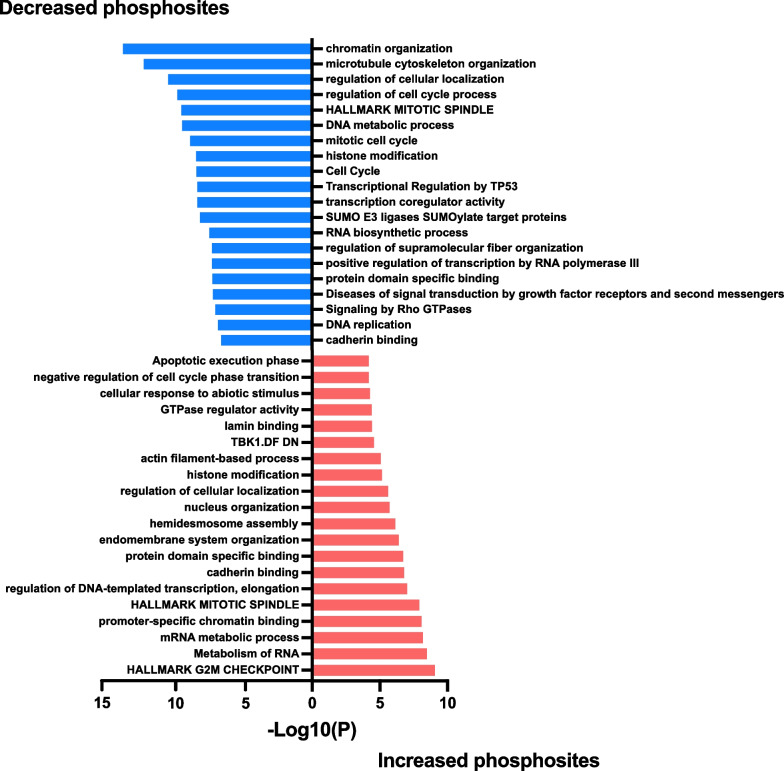
Functional annotation of phosphosites with differential abundance in NEK5-overexpressing and control cells. Data were analysed using Metascape [24]

**Fig. 7:**
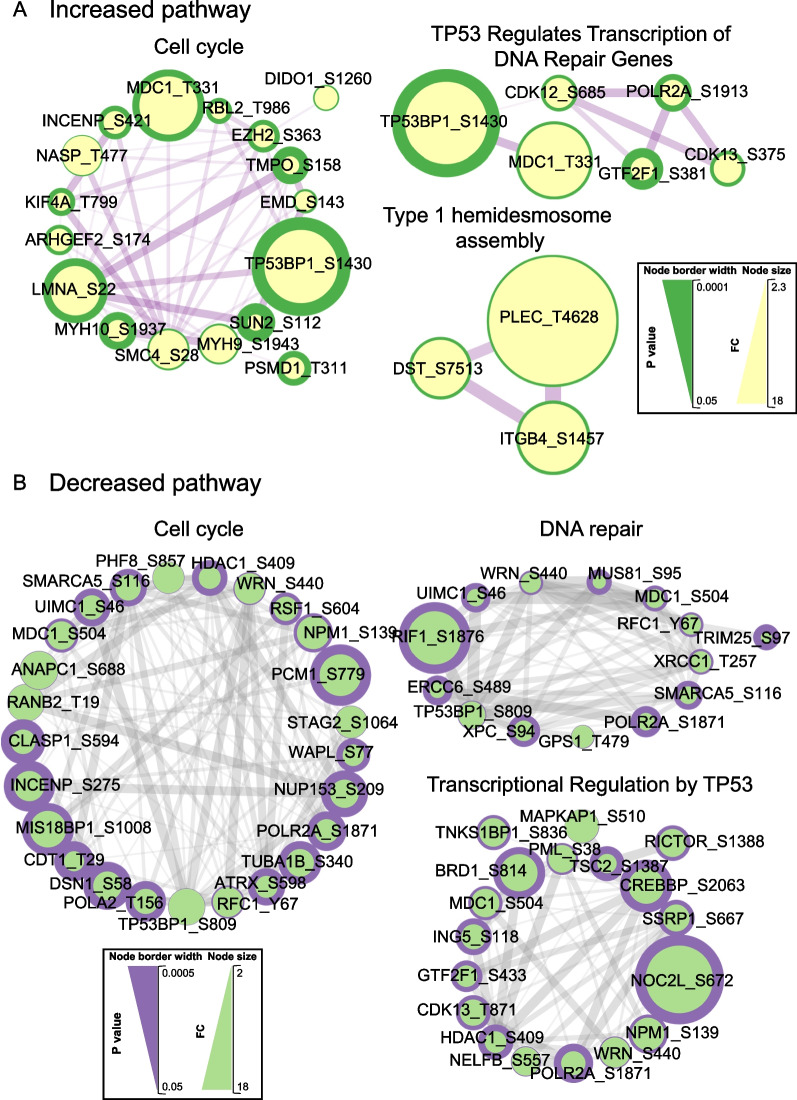
Protein–protein interaction networks for proteins with differentially abundant phosphosites in NEK5-overexpressing and control cells. **A**, **B** Networks of proteins with increased (**A**) and decreased (**B**) phosphosite abundance. Analysis was undertaken using STRING/Cytoscape. Explanation of scales is provided in Fig. 4 legend

Phosphosites exhibiting significantly altered abundance and potentially important functions are highlighted on the volcano plot in Additional file 1: Fig. S5. As for the total proteome, bioinformatic analyses revealed that the cell cycle was a cellular pathway impacted at the phosphoproteomic level, with this pathway and the mitotic spindle enriched in both up- and down-regulated phosphosites (Figs. 6, 7). Amongst the proteins exhibiting altered phosphorylation upon NEK5 overexpression included INCENP, a component of the chromosomal passenger complex, and ANAPC1, a component of the anaphase promoting complex that controls mitotic progression (Fig. 7). Other modulated pathways included TP53-regulated transcription, DNA repair and assembly of hemidesmosomes, protein complexes that mediate association of epithelial cells to the basement membrane (Figs. 6, 7). With regard to the latter pathway, a key protein of interest was ITGB4, which links specific laminins in the extracellular matrix to the intracellular cytoskeleton [48], which exhibited enhanced phosphorylation of S1457.

Integration of the Bio-ID and (phospho)proteomic datasets underscored particular pathways and processes regulated by NEK5. For example, upon integration of the BioID and phosphoproteomic datasets, one protein, SLC16A1, a monocarboxylate transporter, exhibited enrichment in the Bio-ID dataset and SLC16A1 T466 phosphorylation was enhanced upon NEK5 overexpression. However, the amino acid sequence surrounding this site did not exhibit similarity to the NEK5 consensus sequence [49], indicating that it is unlikely to be a direct substrate of NEK5. This integrative approach also identified 11 proteins regulated at the level of both protein expression and site-specific phosphorylation (Table 1). Amongst these were: ARHGEF2, a Rho GEF; TRIP12, an E3 ubiquitin ligase that regulates p19 ARF and the DNA damage response; RFC1 and POLA2, two proteins involved in DNA replication; and CLASP1, a regulator of microtubule dynamics at the mitotic spindle and kinetochore.

**Table 1:** Summary of proteins with significant changes in both protein and phosphosite abundance in NEK5-overexpressing cells

Protein	Protein function	Protein FC	Sites	Site FC
CCDC86	RNA binding	1.38	S21	2.77
			S66	14.64
DTD1	DNA replication	1.76	S194	3.48
			S197	4.27
TRIP12	Ubiquitin fusion, degradation and DNA repair	1.59	S1317	4.15
			S1322	4.15
			S1329	3.13
RIC8A	G-alpha signalling and mitosis	2.38	Y435	3.80
ARHGEF2	Cell motility and cell cycle regulation	1.48	S174	2.97
RFC1	DNA replication	2.22	Y67	0.32
KAT7	DNA replication and transcription	1.56	S100	0.28
			S102	0.28
			S99	0.18
			T104	0.19
			T97	0.19
POLA2	DNA replication	2.52	S147	0.20
			T156	0.20
FOXK1	Transcriptional regulator	1.93	S257	0.19
CLASP1	Microtubule dynamics	1.34	S594	0.16
ZMYM4	Cell morphology and cytoskeletal organisation	0.40	S1064	0.08

## Discussion

A substantial body of evidence links the NEKs to cancer development and progression [10–13, 16, 17, 19]. NEK5, one of the least studied of the family, has recently gained attention due to its potential involvement in breast and prostate cancer [17–19], although the signalling mechanisms and pathways utilized by NEK5 in these contexts remained poorly characterized. In this study we have established a MCF-10A-based model for interrogation of NEK5 function in breast cancer and applied an integrated (phospho)proteomic approach to dissecting NEK5 signalling. Importantly, this identified NEK5-interacting proteins and -regulated pathways consistent with the biological effects of NEK5 overexpression, providing new insights into the oncogenic signalling roles of this kinase.

Consistent with previously reported effects on cell proliferation [19] and cell morphology [18] in breast cancer, overexpression of NEK5 in the MCF-10A system enhanced acinar size in 3D culture, and led to an abnormal, rough acinar morphology. Interestingly, activation of Akt was not altered in the overexpressing cells and that of Erk was decreased, and integration of BioID and (phospho)proteomic analyses revealed that the major impact of NEK5 relating to cell division was not on ‘upstream’ proliferative pathways but instead at the level of microtubule regulation, the mitotic spindle and cell cycle control, consistent with known roles of NEK5 in centrosome separation and spindle formation during mitosis [30]. In addition, we detected pathway alterations that may underpin the effect of NEK5 on acinar morphology, with a significant enrichment for cytoskeletal regulators associated with the RhoH GTPase cycle in the NEK5 interactome, and proteins involved in hemidesmosome assembly prominent amongst those exhibiting enhanced phosphorylation in NEK5-overexpressing cells. In the context of hemidesmosomes, the enhanced phosphorylation of ITGB4 S1457 is of particular note, because serine/threonine phosphorylation of the ITGB4 cytoplasmic tail is known to promote hemidesmosome disassembly and promote cell migration and invasion [50]. Consequently this might contribute to the ‘protrusive’ phenotype of NEK5-overexpressing acini.

A striking and surprising finding from our Western blot analysis of NEK5-overexpressing cells was a significant decrease in Src activation and tyrosine phosphorylation of its downstream substrates FAK and paxillin. Upon interrogation of our proteomic datasets, the most obvious potential link between NEK5 and Src was the identification of CSK as a NEK5 interactor in the BioID screen. CSK phosphorylates the C-terminal tyrosine residue of Src to promote formation of an inactive Src conformation, but to our knowledge, there is no known mechanistic link between NEK5 and CSK, and serine/threonine phosphorylation of CSK is a relatively understudied area [45]. However, our finding suggests a model where NEK5, acting by a phosphorylation-based mechanism and/or by localizing CSK, may positively regulate CSK activity towards Src.

Our integrated proteomic approach also lent further support to relatively new, emerging roles for NEK5. Specifically, our phosphoproteomic analysis identified modulated protein–protein interaction networks associated with DNA repair, which is of interest given the recent demonstration that NEK5 associates with TOPIIβ, a regulator of DNA topology during transcription, and NEK5 regulates DNA damage in response to etoposide [51]. In addition, the presence of multiple proteins associated with mitochondrial organization and gene expression as NEK5 interactors in the BioID screen is consistent with the identification of mitochondrial protein binding partners in a previous MS-based characterization of the NEK5 interactome, including CLPP, that partners CLPX in the CLP protease complex, and demonstration that NEK5 regulates mitochondrial homeostasis and mitochondrial DNA maintenance [20]. In this context, our demonstration of NEK5 interaction with TFAM is supported by Proximity Ligation Assays undertaken by the Kobarg group [20].

Several NEK5-associated pathways identified by our integrated proteomic studies have been linked to other NEK family members. For example, NEK2 has an important role in centrosome separation [30, 52] and is implicated in breast cancer progression [53], while NEK6, NEK7 and NEK9 are involved in mitotic spindle assembly, which requires regulation of microtubule polymerization [54–56]. In addition, most NEK family members have been linked to the DNA Damage Response [14, 57–59]. Finally, other NEK family members are associated with mitochondrial homeostasis and dynamics. NEK1 deficiency is related to abnormal mitochondrial functions and elevated ROS levels [60] while NEK4 deficiency leads to altered mitochondrial morphology and reduced mitochondrial respiration [61]. Consequently, there is overlap in the NEK5-associated pathways identified and those linked to other family members, indicating that that this kinase family must regulate critical biological processes in a concerted and integrated manner.

## Conclusions

Using a powerful new model system for interrogating NEK5 signalling and function in breast cancer, we have determined that NEK5 influences not only breast epithelial cell proliferation but also 3D morphogenesis, and provided novel insights via proteomic interrogation that consolidate the role of NEK5 as a mitotic regulator, lend further support to emerging roles for NEK5 in mitochondrial function and DNA repair, and identify novel functions for this kinase in regulating cytoskeletal organization by Rho GTPases and cell–matrix interaction via hemidesmosomes. Overall this study indicates that NEK5 may play multiple roles in breast cancer development and progression and represents a potential target for therapeutic intervention.

## Supplementary Information

**Additional file 1. Figure S1.** Characterization of the NEK5 interactome via BioID. **A** Schematic of BioID workflow. **B** Volcano plot highlighting proteins with enhanced biotinylation that represent NEK5 interactors. Dotted lines indicate the applied cut-offs of fold change > 2 and *p* < 0.05. Labelled proteins with pink dots are enriched at these cut-offs. Note that these have negative log 2 values. **Figure S2.** Expression of NEK5 and its interactors in breast cancer cell lines. **A** Expression of NEK5 and its relationship to ER status. RNA-seq data deposited in the Cancer Cell Line Encyclopedia (CCLE) and Cancer Dependency Map (DepMap) databases were used to determine the mRNA expression of NEK5 in a panel of 60 breast cancer cell lines, grouped based on ER expression status, and the human immortalized breast epithelial cell line HMEL. Error bars represent mean ± SEM (Standard Error of the Mean), ***Indicates *p* < 0.001 (Student’s t-test). ER, Estrogen receptor; TPM, Transcripts per million. **B** Expression of NEK5 and NEK5 interactors identified by BioID in ER-positive breast cancer cell lines. The Violin plot shows the distribution of expression levels of NEK5 and its interacting proteins based on RNA-seq data deposited in the CCLE and DepMap databases. **Figure S3.** Volcano plot highlighting differentially-expressed proteins in NEK5 overexpressing cells versus MCF-10A control cells. Proteins that are significantly increased or decreased in abundance upon overexpression of NEK5 at cut-offs of FC > 1.5 and *p* < 0.05 are represented by pink and blue dots, respectively. Labelled proteins are the 5 proteins with the largest significant fold changes in either direction and key proteins indicated in the Cytoscape protein-protein interaction networks. **Figure S4.** Validation of MS-based proteomic data. Western blot analysis of MCAM and NCAPD3 expression in control and NEK5-overexpressing MCF-10A cells. The asterisk indicates a non-specific band at 100 kDa. Positions of size markers are indicated. **Figure S5.** Volcano plot highlighting differentially abundant phosphosites in NEK5-overexpressing cells versus MCF-10A control cells. Phosphosites that are significantly increased or decreased in abundance upon overexpression of NEK5 at cut-offs of FC > 1.5 and *p* < 0.05 are represented by pink and blue dots, respectively. Labelled phosphosites are the 5 sites with the largest significant fold changes in either direction and key phosphosites indicated in the Cytoscape protein-protein interaction networks.

**Additional file 2. Table S1.** Results from Bio-ID screen. The table shows the results for proteins with log2 FC > 1 and *p* value < 0.05. **Table S2.** Results from MS-based proteomic analysis. Proteins with FC > 1.5 in either direction and *p* < 0.05 are highlighted by shading. **Table S3.** Results from MS-based phosphoproteomic analysis. Phosphosites with FC > 1.5 in either direction and *p* < 0.05 are highlighted by shading.

## Data Availability

MS data will be made available from the ProteomeXchange Consortium via the PRIDE partner repository. Project accession: PXD035322 (Reviewer account details: Username: reviewer_pxd035322@ebi.ac.uk Password: ebWtPI3o). Other raw data can be obtained from the corresponding author.

## Abbreviations

NEK: Nima-Related Kinases
NEK5: Nima-Related Kinase 5
TNBC: Triple negative breast cancer
EV: Empty vector
MS: Mass spectrometry
BioID: Biotin-labelling identification
