# Supplementary material for: Identification of biological pathways and processes regulated by NEK5 in breast epithelial cells via an integrated proteomic approach
Source: Cell Commun Signal. 2022 Dec 22;20:197. doi: 10.1186/s12964-022-01006-y (PMC9773587; doi:10.1186/s12964-022-01006-y)
Supplement: Supplementary file 2 — Additional file 1. Figure S1. Characterization of the NEK5 interactome via BioID. A Schematic of BioID workflow. B Volcano plot highlighting proteins with enhanced biotinylation that represent NEK5 interactors. Dotted lines indicate the applied cut-offs of fold change > 2 and p < 0.05. Labelled proteins with pink dots are enriched at these cut-offs. Note that these have negative log 2 values. Figure S2. Expression of NEK5 and its interactors in breast cancer cell lines. A Expression of NEK5 and its relationship to ER status. RNA-seq data deposited in the Cancer Cell Line Encyclopedia (CCLE) and Cancer Dependency Map (DepMap) databases were used to determine the mRNA expression of NEK5 in a panel of 60 breast cancer cell lines, grouped based on ER expression status, and the human immortalized breast epithelial cell line HMEL. Error bars represent mean ± SEM (Standard Error of the Mean), ***Indicates p < 0.001 (Student’s t-test). ER, Estrogen receptor; TPM, Transcripts per million. B Expression of NEK5 and NEK5 interactors identified by BioID in ER-positive breast cancer cell lines. The Violin plot shows the distribution of expression levels of NEK5 and its interacting proteins based on RNA-seq data deposited in the CCLE and DepMap databases. Figure S3. Volcano plot highlighting differentially-expressed proteins in NEK5 overexpressing cells versus MCF-10A control cells. Proteins that are significantly increased or decreased in abundance upon overexpression of NEK5 at cut-offs of FC > 1.5 and p < 0.05 are represented by pink and blue dots, respectively. Labelled proteins are the 5 proteins with the largest significant fold changes in either direction and key proteins indicated in the Cytoscape protein-protein interaction networks. Figure S4. Validation of MS-based proteomic data. Western blot analysis of MCAM and NCAPD3 expression in control and NEK5-overexpressing MCF-10A cells. The asterisk indicates a non-specific band at 100 kDa. Positions of size markers are indic [file 12964_2022_1006_MOESM2_ESM.docx]

**Additional File 1: Supplementary Figures**

Identification of biological pathways and processes regulated by NEK5 in breast epithelial cells via an integrated proteomic approach

Camila de Castro Ferezin, Terry C C Lim Kam Sian, Yunjian Wu, Xiuquan Ma, Anderly C. Chüeh, Cheng Huang, Ralf B. Schittenhelm, Jörg Kobarg, Roger J. Daly.


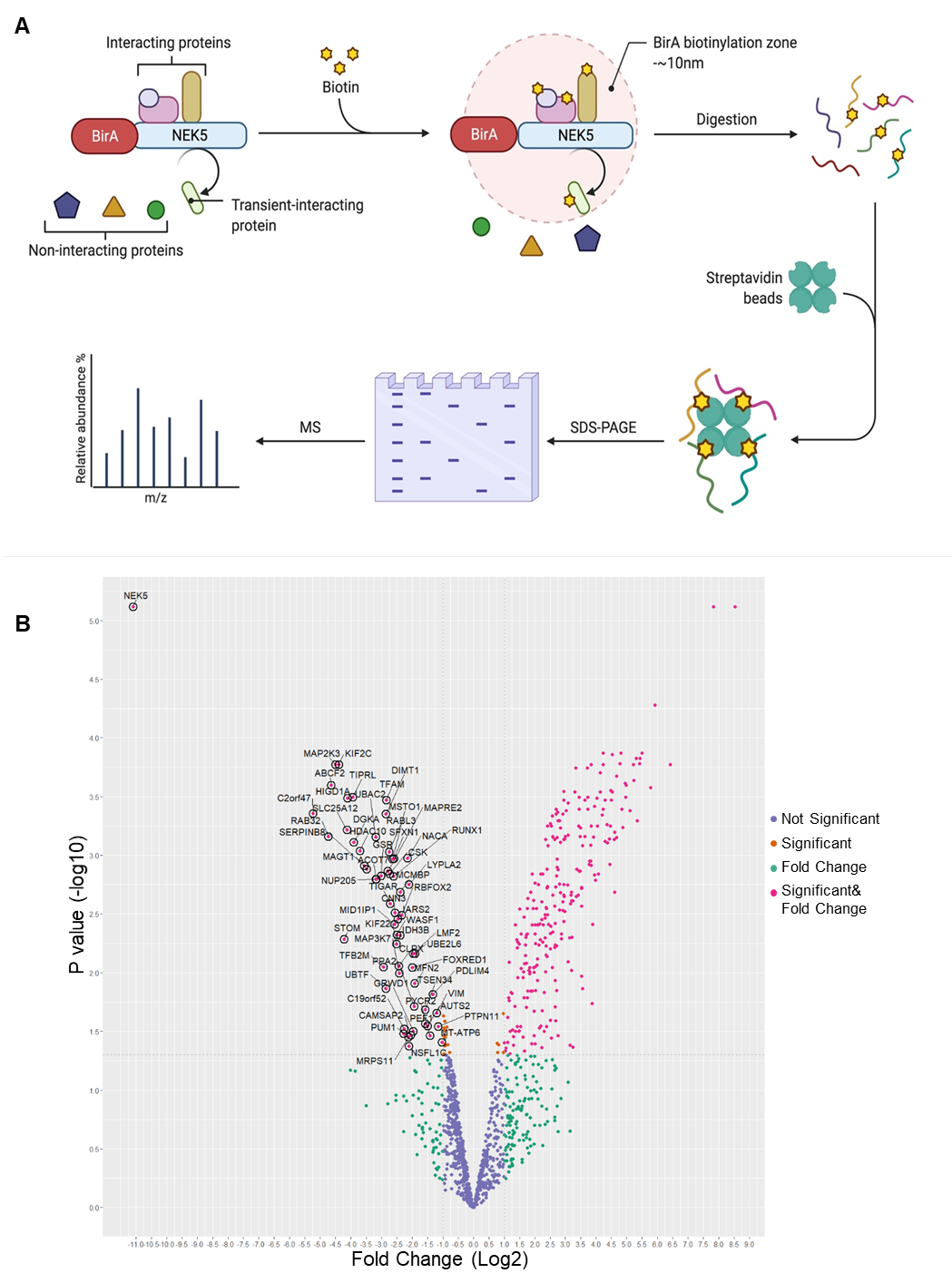


**Figure S1: Characterization of the NEK5 interactome via BioID. A.** Schematic of BioID workflow. **B.** Volcano plot highlighting proteins with enhanced biotinylation that represent NEK5 interactors. Dotted lines indicate the applied cut-offs of fold change >2 and p<0.05. Labelled proteins with pink dots are enriched at these cut-offs. Note that these have negative log 2 values.


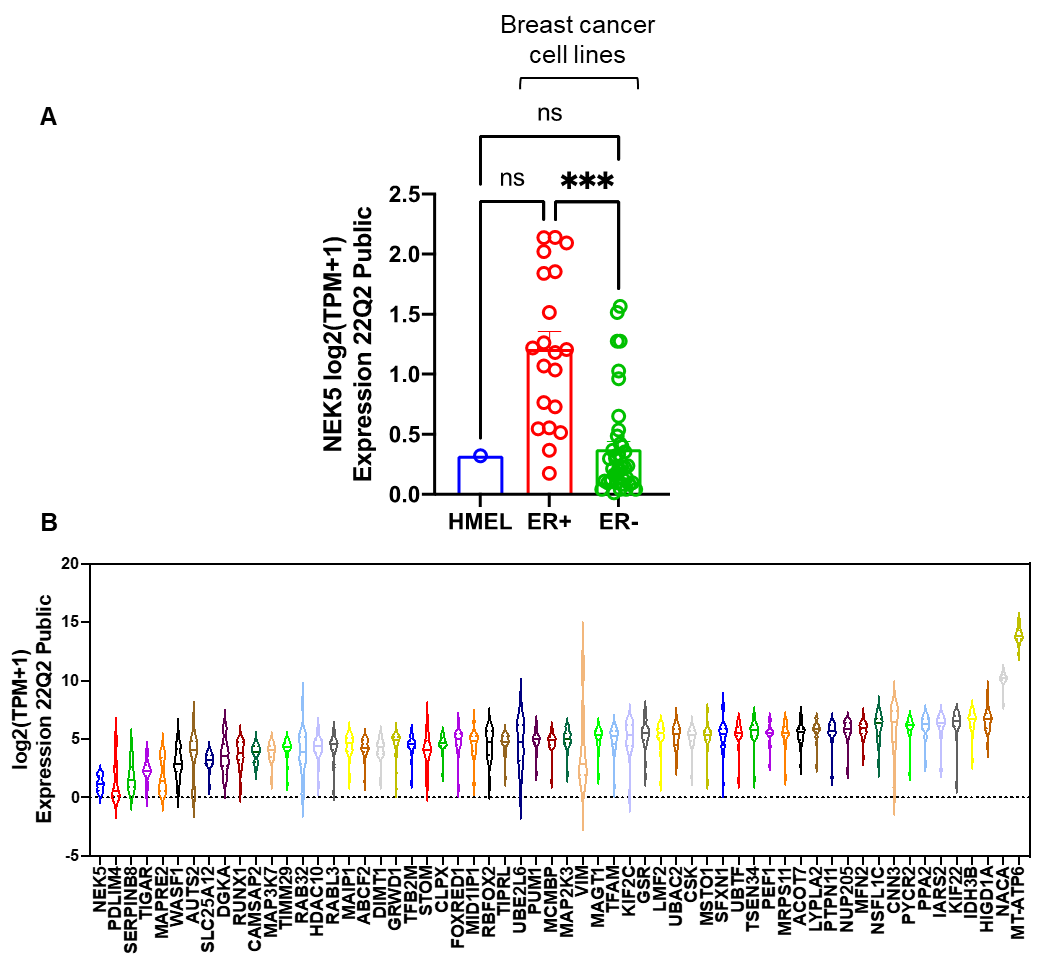


**Figure S2: Expression of NEK5 and its interactors in breast cancer cell lines.** **A.** Expression of NEK5 and its relationship to ER status. RNA-seq data deposited in the Cancer Cell Line Encyclopedia (CCLE) and Cancer Dependency Map (DepMap) databases were used to determine the mRNA expression of NEK5 in a panel of 60 breast cancer cell lines, grouped based on ER expression status, and the human immortalized breast epithelial cell line HMEL. Error bars represent mean ± SEM (Standard Error of the Mean), *** indicates P<0.001 (Student’s t-test). ER, Estrogen receptor; TPM, Transcripts Per Million. **B.** Expression of NEK5 and NEK5 interactors identified by BioID in ER-positive breast cancer cell lines. The Violin plot shows the distribution of expression levels of NEK5 and its interacting proteins based on RNA-seq data deposited in the CCLE and DepMap databases.

**
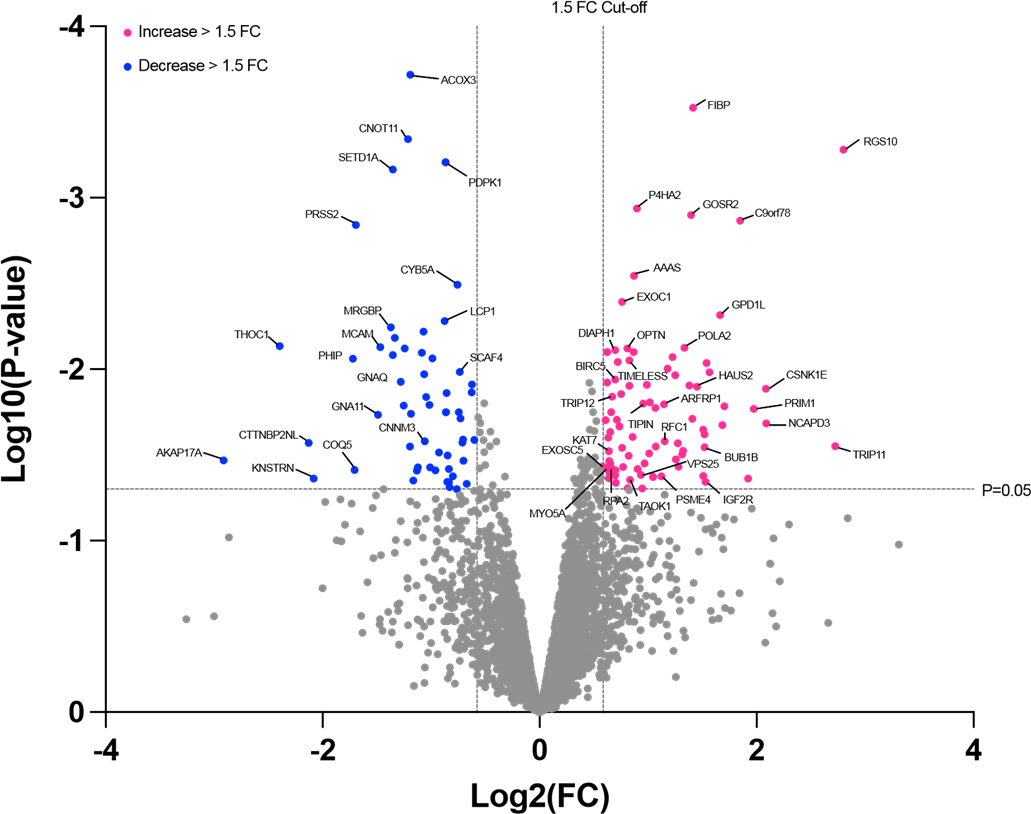
**

**Figure S3. Volcano plot highlighting differentially-expressed proteins in NEK5 overexpressing cells versus MCF-10A control cells.** Proteins that are significantly increased or decreased in abundance upon overexpression of NEK5 at cut-offs of FC>1.5 and p<0.05 are represented by pink and blue dots, respectively. Labelled proteins are the 5 proteins with the largest significant fold changes in either direction and key proteins indicated in the Cytoscape protein-protein interaction networks.


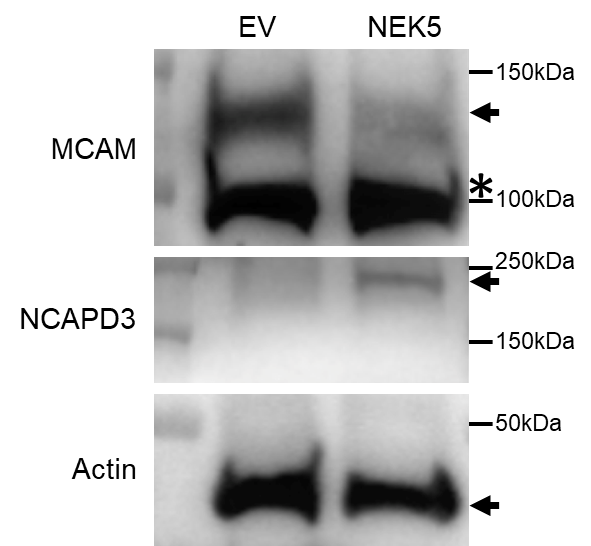


**Figure S4. Validation of MS-based proteomic data.** Western blot analysis of MCAM and NCAPD3 expression in control and NEK5-overexpressing MCF-10A cells. The asterisk indicates a non-specific band at 100 kDa. Positions of size markers are indicated.


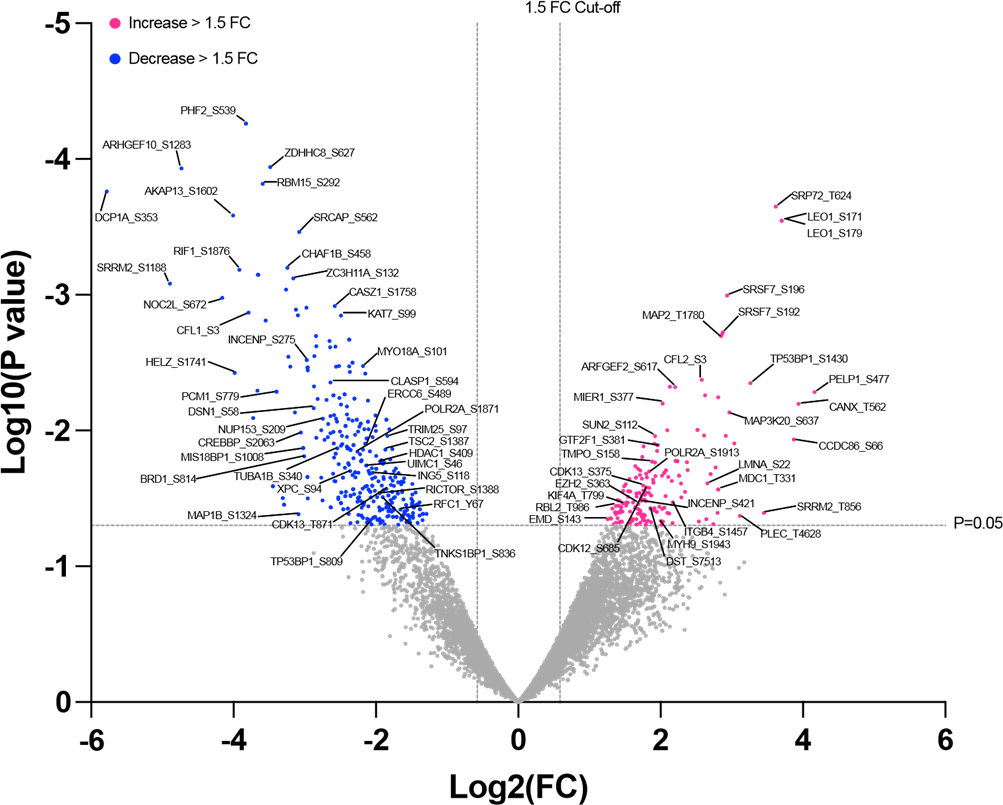


**Figure S5. Volcano plot highlighting differentially abundant phosphosites in NEK5- overexpressing cells versus MCF-10A control cells.** Phosphosites that are significantly increased or decreased in abundance upon overexpression of NEK5 at cut-offs of FC>1.5 and p<0.05 are represented by pink and blue dots, respectively. Labelled phosphosites are the 5 sites with the largest significant fold changes in either direction and key phosphosites indicated in the Cytoscape protein-protein interaction networks.
